# cDNA cloning, characterization and expression analysis of peroxiredoxin 5 gene in the ridgetail white prawn *Exopalaemon carinicauda*

**DOI:** 10.1007/s11033-013-2702-4

**Published:** 2013-10-19

**Authors:** Yafei Duan, Ping Liu, Jitao Li, Jian Li, Baoquan Gao, Ping Chen

**Affiliations:** 1 Key Laboratory of Sustainable Development of Marine Fisheries, Ministry of Agriculture, Yellow Sea Fisheries Research Institute, Chinese Academy of Fishery Sciences, 266071 Qingdao, China; 2 Key Laboratory of South China Sea Fishery Resources Exploitation & Utilization, Ministry of Agriculture, South China Sea Fisheries Research Institute, Chinese Academy of Fishery Sciences, 510300 Guangzhou, China

**Keywords:** *Exopalaemon carinicauda*, Peroxiredoxin 5 (Prx5), Gene cloning, Expression

## Abstract

Peroxiredoxin is a superfamily of antioxidative proteins that play important roles in protecting organisms against the toxicity of reactive oxygen species. In this study, a full-length of peroxiredoxin 5 (designated EcPrx5) cDNA was cloned from the ridgetail white prawn *Exopalaemon carinicauda* by using rapid amplification of cDNA ends (RACE) approaches. The full-length cDNA of the EcPrx5 was of 827 bp, containing a 5′ untranslated region (UTR) of 14 bp, a 3′ UTR of 228 bp with a poly (A) tail, and an open reading frame of 585 bp encoding a polypeptide of 194 amino acids with the predicted molecular weight of 20.83 kDa and estimated isoelectric point of 7.62. BLAST analysis revealed that amino acids of EcPrx5 shared 89, 68, 66, 65, 53 and 51 % identity with that of *Macrobrachium rosenbergii*, *Megachile rotundata*, *Harpegnathos saltator*, *Acromyrmex echinatior*, *Danio rerio*, and *Homo sapiens* counterparts, respectively. The conserved Prx domain and the signature of peroxiredoxin catalytic center identified in EcPrx5 suggested that EcPrx5 belonged to the atypical 2-Cys Prx subgroup. Real time quantitative RT-PCR analysis indicated that EcPrx5 could be detected in all the tested tissues with highest expression level in hepatopancreas. As time progressed, the expression level of EcPrx5 both in hemocytes and hepatopancreas increased in the first 6 h after *Vibrio anguillarum* and white spot syndrome virus challenge, and showed different expression profiles. The results indicated that EcPrx5 involved in immune response against bacterial and viral infection in *E. carinicauda*.

## Introduction

Reactive oxygen species (ROS), such as hydrogen peroxide (H_2_O_2_), superoxide anion and singlet oxygen, are thought to be involved in cancer, aging and various inflammatory disorders [1]. In addition, these ROSs can kill foreign invaders efficiently and also play an important role in immune signal transduction [2, 3]. However, the mass accumulation of ROS in animals will cause serious cell damage, resulting in various diseases [4–6]. To protect themselves against damages of ROS, aerobic organisms have developed a set of antioxidant defense systems, including antioxidant enzymes such as superoxide dismutase, catalase and many kinds of peroxidases [7–9].

Peroxiredoxin (Prx) is a large family of antioxidant proteins ubiquitously found from prokaryotes to eukaryotes [5, 10–12], which play important roles in protecting the organisms against oxidative stress and regulating the intracellular signal transduction [13, 14]. In mammals, six different isoforms of Prx (Prx1-Prx6) have been identified [15, 16]. Based on the number of cysteine residues involved in catalysis and the type of disulfide bond formed, Prxs are divided into three subgroups: 2-Cys (Prx1-Prx4), atypical 2-Cys (Prx5) and 1-Cys (Prx6) [11, 17]. Prx5, also known as PrxV, AOEB166, PMP20 or ACR1, is a mammalian thioredoxin peroxidase that can be addressed to mitochondria, peroxisomes and the cytosol, suggesting that this peroxiredoxin may have an important role as antioxidant in organelles [18, 19]. Several Prx5 have been isolated from vertebrate and invertebrate species [20–23]. However, no studies about Prx5 in *Exopalaemon carinicauda* have been reported till now.

The ridgetail white prawn *E. carinicauda* is an important economical shrimp species naturally distributed in the coasts of Yellow Sea and Bohai Sea, China [24], which contributes to one-third of the gross outcome of the polyculture ponds in eastern China [25, 26]. However, various diseases caused by bacteria and viruses have blossomed within booming *E. carinicauda* cultures, causing economic losses to commercial shrimp aquaculture [26]. Better understanding of the innate immune abilities and immune defense mechanisms of shrimp will be beneficial to the development of health management and disease control in shrimp aquaculture. The aim of this study was to clone the full-length cDNA of Prx5 from hemocytes of *E. carinicauda*, compare its sequence with other known Prx5s from other animals, investigate the expression pattern of EcPrx5 in various tissues, and evaluate its expression in *E. carinicauda* with *Vibrio anguillarum* and white spot syndrome virus (WSSV) challenge.

## Materials and methods

### Animal materials

Healthy adult *E. carinicauda*, averaging weight 1.19 ± 0.32 g, were collected from a commercial farm in Qingdao, China. They were cultured in filtered aerated seawater (salinity 20 ‰, pH 8.2) at 18 ± 0.5 °C for 7 days before processing. There were 30 shrimps in each group. The shrimps were fed daily with a ration of 10 % of body weight, and two-thirds of the water in each group was renewed once daily.

### RNA extraction and cDNA synthesis

Hemocytes were collected with syringe contained an equal volume of anti-coagulant buffer [27], and centrifuged at 800 g, 4 °C for 15 min. Total RNA was extracted from hemocytes using Trizol Reagent (Invitrogen, USA) according to the manufacturer’s instruction. The RNA samples were analyzed in 1.0 % agarose electrophoresis and quantitated at 260 nm, all OD_260_/OD_280_ were between 1.8 and 2.0. The 3′ and 5′ ends RACE cDNA template were synthesized using SMART™ cDNA Kit (Clontech, USA) following the protocol of the manufacturer.

### Cloning the full-length cDNA of EcPrx5

An EST sequences was found in large scale EST sequencing from hemocytes cDNA library of the ridgetail white prawn *E. carinicauda* (GenBank accession no. JK996159), which was constructed using the SMART cDNA library construction kit (Clontech, USA) and have been reported by Duan et al. [24]. Blast analysis showed that they have high similarities with Prx5. According to the EST sequence, a gene specific primer F1 was designed for 3′ RACE, and R1 was designed for 5′ RACE (Table 1).

**Table 1:** Primer sequences used in this study

Primer	Sequence (5′–3′)
F1 (forward)	TGGCGACCATTCTCAGTG
R1 (reverse)	CTTTAGACTTCGGTTCCT
F2 (forward)	AGATTGTTCCACGGTTTTGTG
R2 (reverse)	AATACTTTGCGTCCTGCTGAC
18S-HF	TATACGCTAGTGGAGCTGGAA
18S-HR	GGGGAGGTAGTGACGAAAAAT
UPM	CTAATACGACTCACTATAGGGCAAGCAGTGGTATCAACGCAGAGT CTAATACGACTCACTATAGGGC

Based on the partial sequence data of Prx5, its 3′ and 5′ ends were obtained using SMART RACE cDNA Amplification Kit (Clontech, USA). For 3′ RACE, the PCR reaction was performed using the primer F1 and the anchor primer UPM (Table 1). The PCR reaction systems were 50 μL, including RACE cDNA template 2.5 μL, 10 × Advantage 2 PCR buffer 5 μL, dNTP Mix (10 μmol/L) 1 μL, 50 × Advantage 2 Polymerase Mix 1 μL, primer UPM (10 μmol/L) 5 μL, primer F1 (10 μmol/L) 1 μL, PCR-Grade water 34.5 μL. The PCR reaction conditions were 5 cycles of 94 °C for 30 s, 72 °C for 3 min, 5 cycles of 94 °C for 30 s, 70 °C for 30 s, and 72 °C for 3 min, and 25 cycles of 94 °C for 30 s, 68 °C for 30 s and 72 °C for 3 min. For 5′ RACE, the PCR reaction was performed using the primer R1 and the anchor primer UPM (Table 1). The PCR reaction systems and conditions were the same as those described above.

The PCR fragments were subjected to electrophoresis on 1.5 % agarose gel to determine length differences, and the target band was purified by PCR purification kit (Promega, USA). The purified products were cloned into PMD18-T vector, following the instructions provided by manufacturer (TaKaRa, Japan). Recombinant bacteria were identified by blue/white screening and confirmed by PCR. Plasmids containing the insert were purified (Promega minipreps) and used as a template for DNA sequencing.

### Sequence analysis

The nucleotide and deduced amino acid sequences of EcPrx5 cDNA were analyzed and compared using the BLAST search programs (http://www.blast.ncbi.nlm.nih.gov/Blast.cgi). The multiple sequence alignment of Prx5 amino acid sequences was performed using the programs of Vector NTI advance 10.3 (Invitrogen). A phylogenetic NJ tree of Prxs was constructed by the MEGA 4.0 software [28].

### Tissue expression of EcPrx5

Hemocytes, gill, hepatopancreas, muscle, ovary, eyestalk, stomach and bowel were dissected from unchallenged *E. carinicauda*. The mRNA expressions of EcPrx5 in different tissues were determined by quantitative real-time RT-PCR. Total RNA was extracted as described above. The RNA samples were analyzed in 1.0 % agarose electrophoresis and quantitated at 260 nm, all OD_260_/OD_280_ were between 1.8 and 2.0. Total RNA (5 μg) was reverse transcribed using the PrimeScript™ Real time PCR Kit (TaKaRa, Japan) for real-time quantitative RT-PCR analysis.

### Experimental design of *V. anguillarum* and WSSV challenge

The experiments were divided into the bacterial challenged group, the virus challenged group and the control group. *V. anguillarum* strains was obtained from Germplasm Resources and Genetic Breeding Laboratory, Yellow Sea Fisheries Research Institute, activating on marine agar 2611E. WSSV crude extract were obtained from 10 grams of WSSV-infected tissue from *Litopenaeus vannamei*, which provided from Mariculture Disease Control and Pathogenic Molecular Biology Laboratory, Yellow Sea Fisheries Research Institute, the methods referred to Li et al. [29]. In the experiment, the challenged groups were injected individually with 20 μL live *V. anguillarum* suspended in 0.9 % normal saline (2 × 10^8^ CFU/mL) or 20 μL WSSV crude extract, the control group received individually an injection of 20 μL sterile 0.9 % saline solution. Hemocytes and hepatopancreas of six shrimps from each treatment (the challenged group and the control group) were randomly sampled at 0, 3, 6, 12, 24, 48 and 72 h post-injection respectively, then the samples were snap-frozen in liquid nitrogen. There were three replicates for each time point. Total RNA was extracted and the first strand cDNA was synthesized as described above.

### Expression of EcPrx5 after *V. anguillarum* and WSSV challenge

Real time quantitative RT-PCR was performed on an ABI PRISM 7500 Sequence Detection System (Applied Biosystems, USA) to investigate the expression of EcPrx5. The pair of specific primers F2 and R2 (Table 1) was used to amplify a PCR product of 166 bp. Two primers 18S-HF and 18S-HR (Table 1) were used to amplify an 18S gene of 147 bp as an internal control to verify the successful reverse transcription and to calibrate the cDNA template. The RT-PCR was carried out in a total volume of 20 μL, containing 10 μL SYBR^®^ Premix Ex Taq™ II (2×) (TaKaRa, Japan), 2 μL of the 1:5 diluted cDNA, 0.8 μL each of F2 (10 μmol/L) and R2 primer (10 μmol/L) (or 18S-HF (10 μmol/L) and 18S-HR (10 μmol/L) to amplify the 18S), 0.4 μL ROX Reference Dye II (50 ×)*3 and 6 μL DEPC-treated water. The PCR program was 95 °C for 30 s, then 40 cycles of 95 °C for 5 s and 60 °C for 34 s, followed by 1 cycle of 95 °C for 15 s, 60 °C for 1 min and 95 °C for 15 s. DEPC-treated water for the replacement of template was used as negative control.

RT-PCR data from three replicate samples were analyzed with the ABI 7300 system SDS Software (Applied Biosystems, USA), for estimating transcript copy numbers for each sample. The comparative *C*_T_ method was to analyze the relative expression level of EcPrx5. The *C*_T_ for the target amplified products of EcPrx5 and internal control 18S were determined for each sample. Difference in the *C*_T_ between the target and the internal control, called △*C*_T_, was calculated to normalize the differences in the amount of template and the efficiency of the RT-PCR. In the same challenge time, the △*C*_T_ of the control group was used as the calibrator, and the difference between the △*C*_T_ of the challenged group and the control group was called △△*C*_T_. The expression level of EcPrx5 was calculated by the ${2^{-\vartriangle \vartriangle}}^{C_{\text{T}}}$ comparative *C*_T_ method [30].

Statistical analysis was performed using SPSS software (Ver 11.0). Statistical significance was determined using one-way ANOVA [20] and post hoc Duncan multiple range tests. Significance was set at *P* < 0.05.

## Result

### Sequence characterization of EcPrx5

**Fig. 1:**
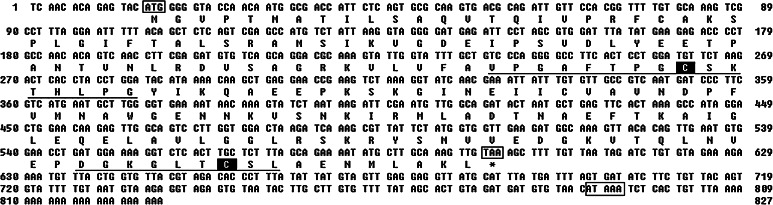
Nucleotide and deduced amino acid sequences of EcPrx5 cDNA of *E. carinicauda*. The *letters in box* indicated the start codon (ATG), the stop codon (TAA) and the polyadenylation signal sequence (ATAAA). The Prx5 signature motifs (VPGAFTPGCSKTHLPG and DGTGLTCSL) were *underlined* and the conserved cysteines were *shaded*

The full-length EcPrx5 cDNA of *E. carinicauda* was obtained by RACE, and the results are shown in Fig. 1. The full-length of EcPrx5 was 827 bp, containing a 585 bp open reading frame (ORF) encoded for 194 amino acids. The cDNA contained a 5′-untranslated region (UTR) of 14 bp, a 3′-UTR of 228 bp including a stop codon (TAA) polyadenylation signal (ATAAA) and a poly (A) tail. The calculated molecular mass was 20.83 kDa, and the estimated isoelectric point was 7.62. The EcPrx5 cDNA sequence has been submitted to the GenBank (GenBank accession number: JX508643).

### Homology analysis of EcPrx5

Sequence analysis with the BLASTP program revealed that the deduced amino acid sequence of EcPrx5 exhibited similarities with Prx5 of invertebrates and vertebrates. It displayed high similarity to Prx5 of *Macrobrachium rosenbergii* (89 %), *Megachile rotundata* (68 %), *Anopheles darlingi* (66 %), *Harpegnathos saltator* (66 %), *Acromyrmex echinatior* (65 %), *Nasonia vitripennis* (64 %), *Tribolium castaneum* (64 %), *Aedes aegypti* (63 %), *Papilio xuthus* (62 %), *Crassostrea gigas* (61 %), *Xenopus laevis* (55 %), *Danio rerio* (53 %), *Bos taurus* (52 %), *Homo sapiens* (51 %), and so on.

**Fig. 2:**
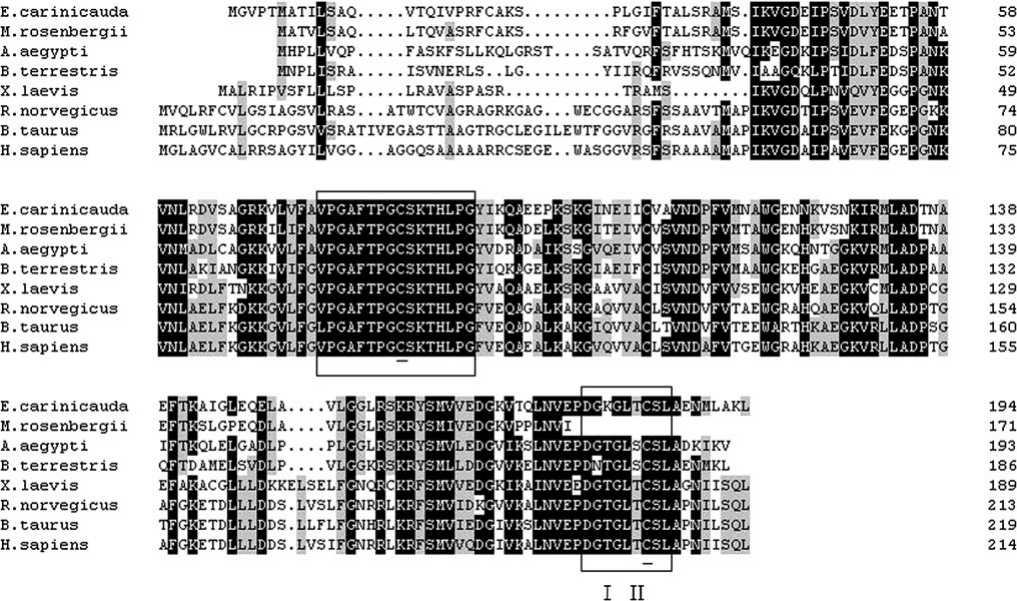
Multiple alignment of EcPrx5 with other known Prx5 s: *M. rosenbergii* (HQ668096), *A. aegypti* (XP_001658149), *Bombus terrestris* (XP_003394825), *X. laevis* (AEM44542), *Rattus norvegicus* (AAH78771), *B. taurus* (AAG53661), *H. sapiens* (CAG33484). Peroxiredoxins signatures VPGAFTPGCSKTHLPG and DGTGLTCSL were marked by frame and the two highly conserved amino acids were indicated by *solid lines*

Multiple sequence alignment revealed that the cysteine residue (Cys^83^ and Cys^184^) was conserved in all the analyzed Prx5 s. In addition, Cys^83^ and Cys^184^, positioned within VPGAFTPGCSKTHLPG and DGTGLTCSL respectively, were deduced to form the intramolecular disulfide bond. No signal peptide was identified by the signalP program (Fig. 2).

**Fig. 3:**
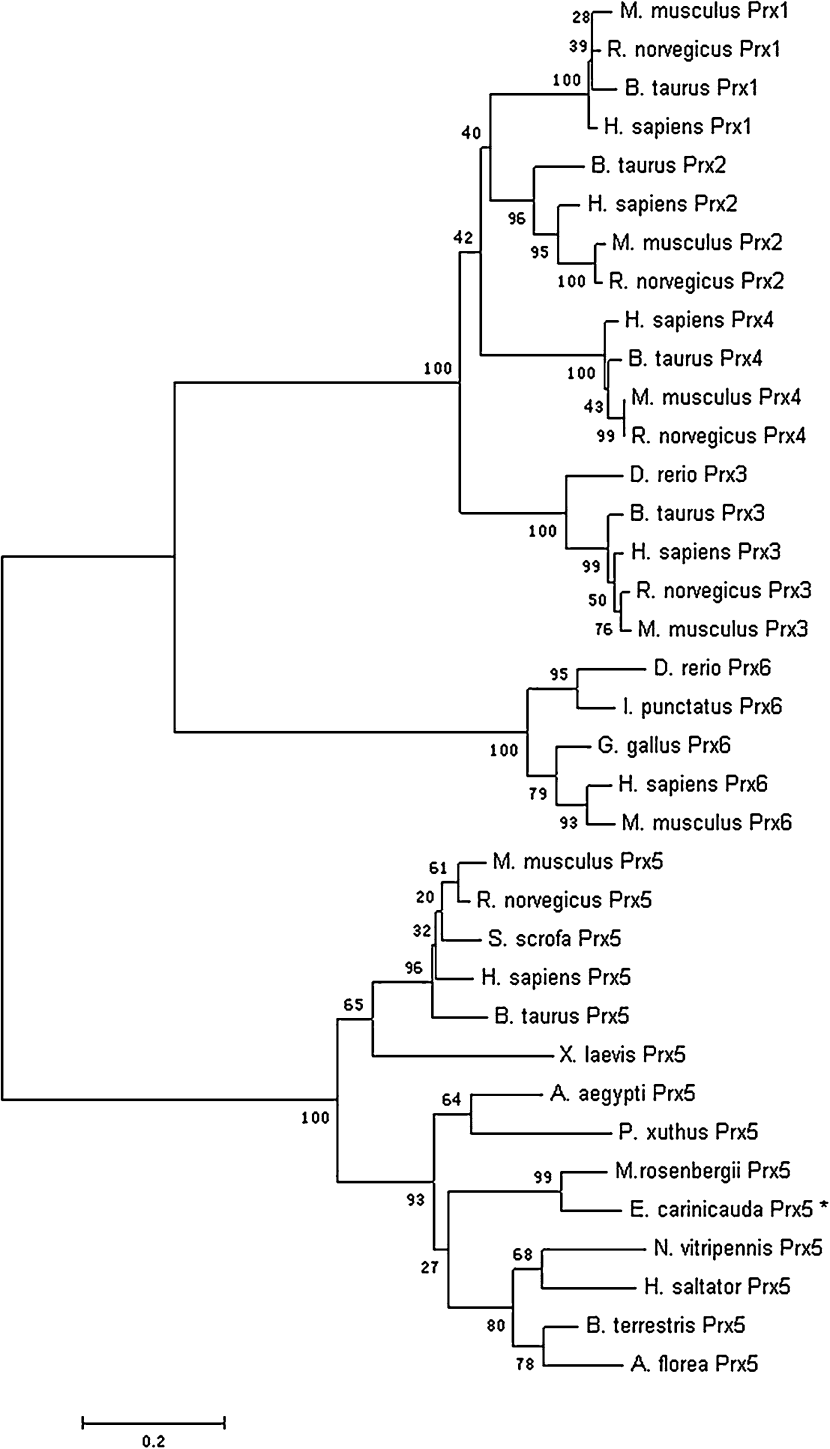
Phylogenetic tree of different species Prxs on the basis of the amino acid sequence using neighbor-joining distance analysis. The numbers at the forks indicated the bootstrap. The protein sequences used for phylogenetic analysis were as follows: Prx1 from *H. sapiens* (CAG28580), *Mus musculus* (CAM16508), *B. taurus* (NP_776856) and *R. norvegicus* (NP_476455), Prx2 from *H. sapiens* (CAG46588), *M. musculus* (AAH86783), *B. taurus* (NP_777188) and *R. norvegicus* (NP_058865), Prx3 from *H. sapiens* (CAG29340), *M. musculus* (AAH05626), *B. taurus* (NP_776857), *R. norvegicus* (EDL94585) and *D. rerio* (NP_001013478), Prx4 from *H. sapiens* (CAG46506), *M. musculus* (CAM23141), *B. taurus* (NP_776858) and *R. norvegicus* (NP_445964), Prx5 from *H. sapiens* (CAG33484), *M. musculus* (AAG13450), *B. taurus* (AAG53661), *Sus scrofa* (NP_999309), *R. norvegicus* (AAH78771), *X. laevis* (AEM44542), *M. rosenbergii* (HQ668096), *N. vitripennis* (XP_001603445), *A. aegypti* (XP_001658149), *B. terrestris* (XP_003394825), *H. saltator* (EFN85437), *Apis florae* (XP_003694601) and *P. xuthus* (BAM18222), Prx6 from *H. sapiens* (NP_004896), *M. musculus* (NP_031479), *Gallus gallus* (NP_001034418), *D. rerio* (NP_957099) and *Ictalurus punctatus* (ABG77029)

A molecular phylogenetic tree was constructed to further analyze the evolutionary relationships among animal Prx sequences (Fig. 3). Base on MEGA 4.0 analysis, the Prxs were identified with three distinct clades, 1-Cys, 2-Cys, and atypical 2-Cys. All the Prx5s formed the atypical 2-Cys subgroup, and all the Prx6s were clustered together and formed a sister group with Prx5 s. Prx1, Prx2, Prx3 and Prx4 clustered into a branch and constituted to 2-Cys subgroup. EcPrx5 was clustered into atypical 2-Cys subgroup. In the Prx5s subgroup, the sequences from vertebrate diverged from invertebrate, and EcPrx5 was placed in the invertebrate branch with other arthropods.

### Tissue expression of EcPrx5

**Fig. 4:**
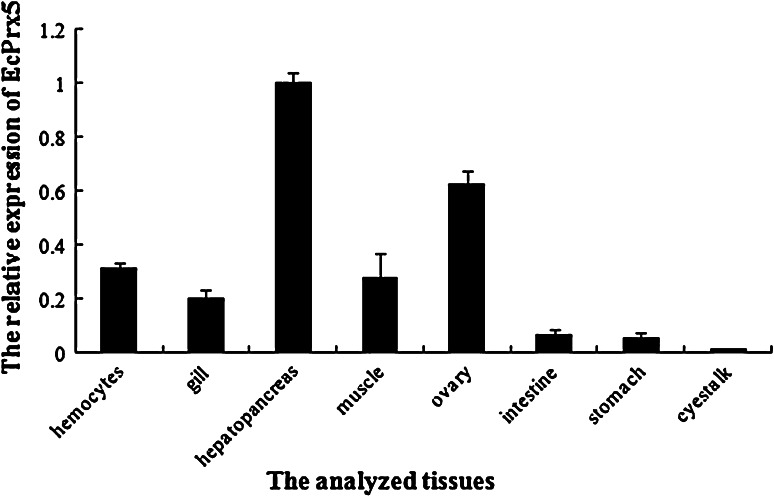
Tissue specific expression of EcPrx5 mRNA related to hepatopancreas expression by the real-time PCR. The reference gene is 18S. *Vertical bars* represent the mean ± SD (*N* = 3)

Quantitative real-time RT-PCR was employed to investigate the distribution of EcPrx5 mRNA in different tissues. The mRNA transcripts of EcPrx5 could be detected in all the examined tissues with different expression levels including hemocytes, gill, hepatopancreas, muscle, ovary, intestine, stomach and eyestalk (Fig. 4). The highest expression was found in hepatopancreas, and the lowest was in eyestalk.

### EcPrx5 gene expression after *V. anguillarum* and WSSV challenge

The mRNA expression levels of EcPrx5 in hemocytes and hepatopancreas of *E. carinicauda* after *V. anguillarum* and WSSV challenge were quantified by real-time RT-PCR with 18S gene as internal control. For both EcPrx5 and 18S genes, there were only one peak at the corresponding melting temperature in the dissociation curve analysis, indicating that the PCR was specifically amplified.

**Fig. 5:**
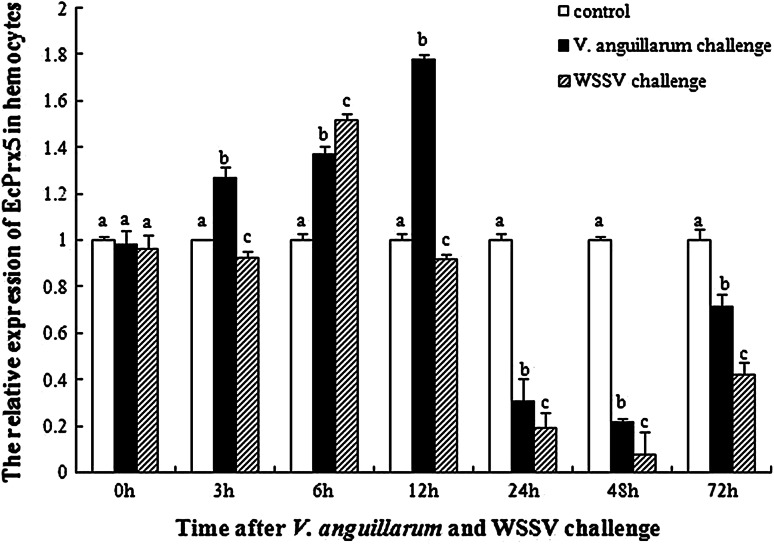
The EcPrx5 mRNA expression levels relative to 18S mRNA levels analyzed by real-time PCR in haemocytes of *E. carinicauda* at different time intervals after *V. anguillarum* and WSSV challenge treatment. *Vertical bars* represent the mean ± SD (*N* = 3). Data without shared letters were significantly different (*P* < 0.05) among treatments in the same exposure time

The expression levels of EcPrx5 in ridgetail white prawn hemocytes after *V. anguillarum* and WSSV challenge were shown in Fig. 5. Compared to the control, the expression of EcPrx5 in *V. anguillarum* and WSSV challenged group increased significantly and reached to the maximum at the first 12 and 6 h after challenge respectively, which was 1.78 and 1.52-fold respectively of that in the control group (*P* *<* 0.05). Afterwards, the expression of EcPrx5 in the two groups decreased gradually, and dropped to the lowest level at 48 h after challenge, which was only 0.22 and 0.08-fold respectively of that in the control group (*P* *<* 0.05), then recovered to 0.72 and 0.42-fold respectively of the control at 72 h after challenge.

**Fig. 6:**
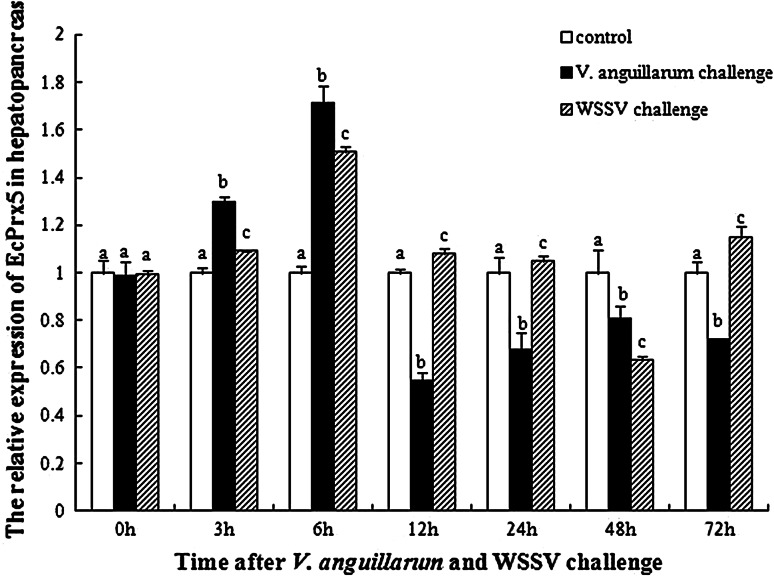
The EcPrx5 mRNA expression levels relative to 18S mRNA levels analyzed by real-time PCR in hepatopancreas of *E. carinicauda* at different time intervals after *V. anguillarum* and WSSV challenge treatment. *Vertical bars* represent the mean ± SD (*N* = 3). Data without shared letters were significantly different (*P* < 0.05) among treatments in the same exposure time

Expression profiles of EcPrx5 in hepatopancreas after *V. anguillarum* and WSSV challenge was shown in Fig. 6. Compared to the control, the EcPrx5 mRNA expression levels of *V. anguillarum* and WSSV challenged groups increased significantly and reached the highest at 6 h after challenge, which was 1.71 and 1.51-fold respectively of that in the control group (*P* *<* 0.05). After 6 h challenge, EcPrx5 mRNA expression levels in *V. anguillarum* challenged group decreased gradually and reached to a low level at 12 h after challenge (0.54-fold of that in control group, *P* *<* 0.05), then recovered to 0.71-fold of the control at 72 h after challenge. However, the EcPrx5 mRNA expression levels of WSSV challenged group decreased and reached the lowest at 48 h after challenge (0.63-fold of that in control group, *P* *<* 0.05), then rised gradually and recovered to 1.14-fold of the control at 72 h after challenge.

## Discussion

The peroxiredoxin family is an evolutionarily conserved group of antioxidants that protect cells from oxidative damage by catalyzing the reduction of a wide range of cellular peroxides [31]. In the present study, a novel Prx gene (EcPrx5) was cloned from *E. carinicauda*. Prior to this study, no full-length cDNA of Prx5 has been isolated in *E. carinicauda*, and our study is the first report on cloning of the full-length cDNA of Prx5 in *E. carinicauda*. Homology analysis revealed that the deduced amino acid sequence of EcPrx5 had more than 50 % similarity with Prx5 of other animals (89 % with *M. rosenbergii*, 68 % with *M. rotundata*, 66 % with *H. saltator*, 65 % with *A. echinatior*, 53 % with *D. rerio* and 51 % with *H. sapiens*). Phylogenetic analysis showed a closer relationship of EcPrx5 with other animals, indicating that the EcPrx5 gene belonged to the atypical 2-Cys Prx subgroup. Multiple sequence alignment analysis revealed that two cysteines (Cys^83^ and Cys^184^) are highly conserved in all of the Prx5 investigated, which located in the Prx signature motifs, and critical for enzyme function serving as a catalytic site and a resolving residue [20]. Compared to Prx5 s of other animals, the Prx signature motifs VPGAFTPGCSKTHLPG appeared to be well conserved, while the motifs DGTGLTCSL had obvious difference. At position I, threonine was replaced by lysine in *E. carinicauda*, and threonine is substituted for serine in insects at position II, the reason would be study in the future.

Quantitative real-time RT-PCR revealed that EcPrx5 was expressed in all the tested tissues, and the highest expression level occurred in the hepatopancreas. As reported by Maningas et al. [32], the differential expression of Prx in various shrimp tissues shows that it is an important molecule that could effectively be involved in a number of physiological activities. Therefore, the EcPrx5 expressed in different tissues of *E. carinicauda* indicated that it could potentially be involved in different physiological process, such as ROS clearance [33, 34], cell differentiation [35, 36], proliferation [37], apoptosis [38], signal transduction [39], immune response [20, 21], and so on.

Various diseases, which mostly caused by bacteria and viruses, have affected the commercial shrimp aquaculture. Prx have been proposed to play a part in the physiological oxidative stress response to bacterial and viral infections in arthropods. Information about the expression profile of Prx5 after bacterial and viral challenge would be helpful in understanding its biological function. *V. anguillarum* and WSSV are both the extremely virulent pathogen prevalent causing mass mortalities and economic losses in shrimp aquaculture [40–42]. When pathogens enter into the body of the shrimp, they will encounter the innate immune systems [43] and ROS are released by oxidative stress in response to them. In the present study, live *V. anguillarum* and WSSV were chosen for challenging the shrimp, so that the shrimp health condition could be affected severely by the production of *V. anguillarum* and WSSV. The level of Prx5 transcripts in hemocytes of *Argopecten irradians* was up-regulated and appeared to be time-dependent after *V. anguillarum* challenged, which indicated that Prx5 is a inducible protein that plays an important role in the immune response against bacterial infection [20]. It reported that in addition to the general antioxidant role of Prx, it may also be associated with immune responses, where Prx could serve to remove ROS [44]. So, the provoked increase of Prx5 expression level was found as one of protection approaches of organisms from further damage.

In our study, EcPrx5 expression in *E. carinicauda* hemocytes and hepatopancreas was up-regulated at earlier time and then decreased gradually with *V. anguillarum* and WSSV challenge. The results showed that EcPrx5 might be involved in a transient systemic immune response to the *V. anguillarum* and WSSV stimulation. Prx5 gene expression was significantly up-regulated until 12 h p.i in gills and then down-regulated in the following p.i. time points at 24 and 48 h in IHHNV infected *M. rosenbergii* [16], which were consistent with our results. After *V. anguillarum* and WSSV challenged 6 h, the transcripts level of EcPrx5 in hemocytes and hepatopancreas of both the challenged group were significant higher than that in the control group, it can be deduced that the challenged shrimps would generate a mass of ROS which need to be eliminated by extra Prx translated from more Prx transcripts. As time progressed, the expression of EcPrx5 in hemocytes dropped to a low level at 24 and 48 h in both *V. anguillarum* and WSSV challenged group, which might because of the infection progress brought more bacterias and virus, and destroyed severely to the normal function of shrimp’s cells and finally caused that the expression of EcPrx5 in the challenged group decreased gradually [7]. EcPrx5 showed a different expression profile in hemocytes and hepatopancreas after *V. anguillarum* and WSSV challenge, this might be caused by the different function of hemocytes and hepatopancreas in the immune defense system. It has been proved that hemocytes are key cells for invertebrate’s innate defense reactions [45, 46] and play an important role in the host immune functions when the organism is attacked by bacterias or viruses [47, 48]. This study showed that EcPrx5 may serve to decrease the cellular damage caused by *V. anguillarum* and WSSV, and the knowledge on this gene expression studies can provide useful tools in understanding and quantifying how these organisms respond to various biotic environmental stress.

In conclusion, a novel Prx cDNA (EcPrx5) was cloned from *E. carinicauda*, and it constitutively expressed in the tissues of hemocytes, gill, hepatopancreas, mucle, ovary, intestine, stomach and eyestalk. The rapid and dynamic expression profiles in hemocytes and hepatopancreas challenged with *V. anguillarum* and WSSV indicated that EcPrx5 was perhaps involved in the immune response against bacterial and viral infection. However, further work is required to better understand the regulation of antioxidant enzymes under oxidative stresses.
